# Immunostimulatory Effects of Korean Mineral-Rich Seawaters on Cyclophosphamide-Induced Immunosuppression in Mice

**DOI:** 10.3390/md22060234

**Published:** 2024-05-21

**Authors:** Choong-Gon Kim, Jae Ho Choi, Sae-Kwang Ku, Chang-Hyun Song

**Affiliations:** 1 Marine Ecosystem Research Center, Korea Institute of Ocean Science and Technology, Busan 49111, Republic of Korea; kimcg@kiost.ac.kr (C.-G.K.); jeaho719@kiost.ac.kr (J.H.C.); 2 Ocean Science and Technology School, Korea Maritime and Ocean University, Busan 49112, Republic of Korea; 3 Department of Anatomy and Histology, College of Korean Medicine, Daegu Haany University, Gyeongsan 38610, Republic of Korea

**Keywords:** deep seawater, deep ocean water, micronutrient, mineral, immunomodulation, immunostimulant, immune deficiency

## Abstract

Deep seawater (DS), obtained from a depth over 200 m, has health benefits due to its rich nutrients and minerals, and intake of DS has shown diverse immunomodulatory effects in allergies and cancer. Therefore, the immunostimulatory effects of Korean mineral-rich seawaters were examined in a cyclophosphamide (CPA)-induced immunosuppression model. Three samples of Korean seawater, namely DS from the East Sea off the coasts of Pohang (PDS) and Uljin (UDS), and seawater from the West Sea off the coast of Boryeong (BS), were collected. The seawaters were abundant in several minerals (calcium, iron, zinc, selenium, etc.). Mice were orally administered the seawaters for 42 days, followed by CPA-induced immunosuppression. The CPA induction reduced the weight of the spleen and lymph nodes; however, the administration of seawaters increased the weight of the lymphoid organs, accompanied by stimulation of natural killer cells’ activity and NF-kB-mediated cytokine production (IFNγ, TNFα, IL1β, IL6, and IL12). The mouse-derived splenocytes showed lymphoproliferation without cytotoxicity in the seawater groups. Histopathological analysis revealed that the seawaters improved the CPA-induced atrophic changes by promoting lymphoproliferation in the spleen and lymph nodes. These results provide useful information for the use of Korean mineral-rich seawaters, particularly PDS and UDS, as alternative immunostimulants under immunosuppressive conditions.

## 1. Introduction

The immune system plays a crucial role in the host’s defense against foreign intruders and cancers [1]. It comprises two fundamental subsystems: innate and adaptive immunity. Innate immunity serves as the first line of defense, offering an immediate immune response without immunologic memory, whereas adaptive immunity provides an antigen-specific response with the capacity for memory to facilitate rapid and enhanced responses upon subsequent exposures to antigens [1]. Both immune cells function complementarily through a complex interplay with soluble mediators (e.g., cytokines, growth factors etc.), leading to productive immune responses [2]. However, immune functions can decline due to aging, chronic illnesses, stress, and insufficient nutrients [3], and defects in either immune system can provoke immune suppression or the host’s vulnerability [4]. Immunostimulants are generally prescribed to enhance the immune response in infections, cancers, and immunodeficiencies [5]. They include bacterial products, carbohydrates (e.g., glucans, trehalose, etc.), synthetic drugs (e.g., levamisole, thalidomide, lenalidomide, etc.), and recombinant cytokines (e.g., interferon, interleukin 2, etc.). However, the induced immunostimulation can lead to toxicities involved in the acute phase of the response with leukocytosis and fever, cell and tissue injury, and overproduction of cytokines. Furthermore, the synthetic agents may have severe side effects such as granulocytopenia in levamisole, birth defects in thalidomide and lenalidomide, and “vascular leak” syndrome in recombinant interleukin (IL)2 [5,6]. Certain natural nutrients have shown a crucial role in maintaining optimal immune responses and improving immune deficiency, along with their safety profiles for clinical application [6,7]. Hence, exploring safe and effective immunostimulants from natural products is currently of global interest.

Micronutrients (e.g., vitamins and minerals) are essential for acting as immunomodulators and protecting the host’s immune response. Micronutrient deficiencies are the most prevalent type of nutritional disorders, affecting more than two billion people worldwide [8]. Micronutrient inadequacy or deficiency is associated with impaired immunity, and the immunostimulatory roles of micronutrients have been well established in previous studies [7,8]. Minerals fulfil a wide variety of immunomodulatory functions despite being present in trace amounts, constituting about 4–6% of the human body, while any deficiencies in minerals could temporarily induce immune suppression or even immune dysregulation in the long term [9,10]. For instance, zinc (Zn) and selenium (Se) deficiencies may suppress the activities of both innate and adaptive immunity; iron (Fe) deficiency may impair T cells’ function and B cells’ proliferation; and copper (Cu) deficiency may lead to reduced humoral and cellular immune function [6,7,11]. In addition, since Zn, Se, Fe, and Cu have antioxidant activities, their deficiencies can increase oxidative stress in immune cells [6,12,13]. Mineral deficiency is widespread globally, and it can occur even in people with a well-balanced diet [11]. The global prevalences of the risk of calcium (Ca) and Zn deficiency in 2011 were reported to be 3.5 billion and 1.1 billion people, respectively, and cases of Fe deficiency-related anemia alone in 2016 exceeded 1.2 billion people [14,15]. The risk of mineral deficiency is especially increasing in patients with chronic diseases, older individuals, vegetarians, and pregnant women [11]. Many other minerals have benefits in maintaining normal immune responses, particularly trace elements, the details of which remain to be elucidated.

Deep seawater (DS) is a natural resource obtained from a depth of over 200 m. It is characterized by low-temperature stability, high purity, and high contents of minerals such as potassium (K), magnesium (Mg), chromium (Cr), fluoride (F), boron, iodide, molybdenum, and vanadium, as well as Cu, Fe, Se, and Zn [13]. Intake of DS has shown diverse immunomodulatory effects in patients with atopic eczema/dermatitis syndrome and allergic rhinitis, and in animal model of *Helicobacter pylori* infection, with no adverse effects even with long-term consumption [16,17,18,19]. DS-based drinking water has also been reported to have potential therapeutic effects in several disease models, including cancer, obesity, hyperlipidemia, diabetes, and hypertension (reviewed in [13]). This suggests that DS can be a good source for supplying the essential minerals involved in various potential therapeutic uses, including immunomodulation. Previously, we demonstrated that balneotherapy in mineral-rich seawaters, including DS in Korea, had antiallergic effects in an atopic dermatitis animal model [20]. Thus, this study aimed to examine the immunostimulatory effects of Korean mineral-rich seawaters on cyclophosphamide (CPA)-induced immunosuppression.

## 2. Results

### 2.1. Mineral Elements Contained in Korean Seawaters

Two samples of Korean DS were collected at depths of 200 m and 250 m in the East Sea, located 32 km off the coast of Pohang (PDS) and 61 km off the coast of Uljin (UDS), respectively. Another seawater sample was collected at a depth of 50 m in the West Sea, located 55 km off the coast of Boryeong (BS). Forty-two minerals were examined in the seawaters, and those with a content of more than 0.5 ppb (μg/L) are listed in Table 1. The mineral amounts were mostly similar among the seawaters, and the minerals were divided into major and trace elements based on an amount of 0.1 ppm (mg/L). The major elements of the three seawaters consisted mainly of sodium (Na), chloride (Cl^−^), and sulfate, accounting for approximately 32%, 58%, and 6% of the total minerals, respectively, and the other major elements accounted for about 3.8–3.9%. The trace elements accounted for only about 0.02%. The minerals showing differences of more than 10% in their amounts among the seawaters were as follows: compared with BS, PDS contained more Cu (47%), Ca (14%), Cr (37%), and Zn (19%), while UDS contained more Ca (10%), Cr (38%), and Zn (14%). Conversely, BS contained more F (12%), aluminum (67% and 29%), and barium (59% and 50%) compared with PDS and UDS, respectively. Silver, uranium, lead, cesium, and tungsten were present in amounts less than 0.5 ppb, and beryllium, zirconium, cadmium, mercury, bismuth, niobium, indium, tantalum, and rhenium were not detected. The hardness was 350, 352, and 340 mg/L in BS, PDS, and UDS, respectively.

### 2.2. Changes in Body Weight and Weight of Lymphoid Organs

**Figure 1:**
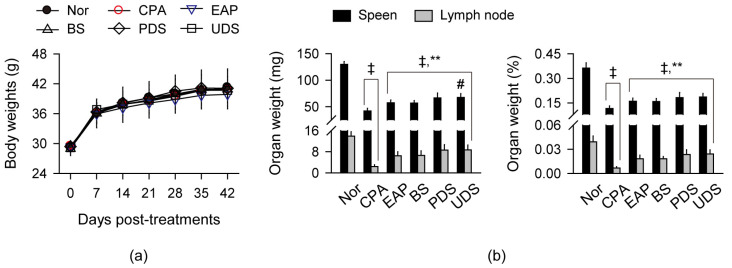
Changes in body weight and the weight of lymphoid organs. (**a**) Body weight changes in the cyclophosphamide (CPA)-induced immunosuppressive model after oral administration of Korean seawaters. The CPA model was administered an exopolymer from *Aureobasidium pullulans* SM-2001 (EAP) or three samples of Korean seawaters, which were deep seawaters from off the coasts of Pohang (PDS) and Uljin (UDS), and seawater from off the coast of Boryeong (BS). (**b**) Weight of the spleen and lymph nodes. Values are expressed as the means ± standard deviations (SDs) in 10 samples. ‡: *p* < 0.01, compared with the normal control (Nor); **: *p* < 0.01, compared with the CPA model control (CPA); #: *p* < 0.05, compared with the BS group.

Mice received an oral administration of the seawaters diluted to 9% in distilled water (DW) to contain 0.9% sodium chloride (NaCl) for 42 days, and immunosuppression was induced by CPA (Figure 1). An exopolymer from *Aureobasidium pullulans* SM-2001 (EAP) was used as a positive control for immunostimulation [21,22]. There were no differences in the changes in body weight among all groups regardless of the treatments (Figure 1a). However, the absolute weight of the lymphoid organs, spleen, and lymph nodes, and their relative weight to body weight were reduced in the CPA control group compared with the normal control (*p* < 0.01), while they were significantly increased in the treatment groups of EAP, BS, PDS, and UDS compared with the CPA control (*p* < 0.01, Figure 1b). The absolute weight of the spleen was notably increased in the UDS group compared with the BS group (*p* < 0.01).

### 2.3. Effects on Cell Viability and Proliferation in Primary Splenocytes Ex Vivo

**Figure 2:**
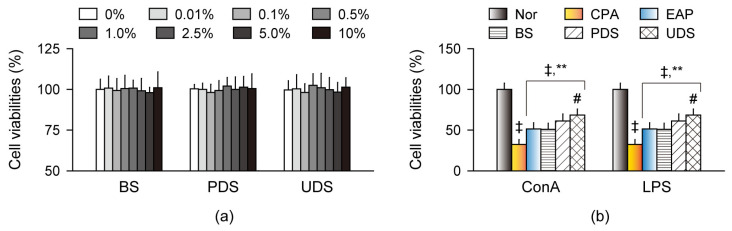
Effects on cell viability and proliferation in primary splenocytes ex vivo. (**a**) Cell viability in the normal control (Nor)-derived splenocytes in the treatments with the Korean seawaters. Three samples of Korean seawaters, namely deep seawaters from off the coasts of Pohang (PDS) and Uljin (UDS), and seawater from off the coast of Boryeong (BS), were given at the indicated concentrations. (**b**) Concanavalin A (ConA)- and lipopolysaccharide (LPS)-induced lymphoproliferation in the splenocytes derived from the cyclophosphamide (CPA)-induced model groups. The CPA model was administered an exopolymer from *Aureobasidium pullulans* SM-2001 (EAP) or three samples of Korean seawaters (BS, PDS, and UDS). Values are expressed as the means ± SDs in six independent experiments. ‡: *p* < 0.01, compared with the Nor group; **: *p* < 0.01, compared with the CPA model control (CPA); #: *p* < 0.05, compared with the BS group.

Immune cell cytotoxicity was examined in the mouse-derived splenocytes, and lymphoproliferation was assessed in the primary splenocytes treated with concanavalin A (ConA) and lipopolysaccharide (LPS), known as a T cell division promoter and a B cell activator, respectively (Figure 2) [22,23]. Cell viabilities were not different in the normal control-derived splenocytes after treatment with the BS, PDS, and UDS at seven dose ranges of 0.1% to 10% compared with the non-treatment control (Figure 2a). The ConA- and LPS-induced lymphoproliferation was significantly lower in the splenocytes isolated from the CPA control group than in those from the normal control; however, they were higher in the splenocytes isolated from the EAP, BS, PDS, and UDS groups compared with those from the CPA control (*p* < 0.01, Figure 2b). Furthermore, the lymphoproliferative effects were significantly higher in splenocytes of the UDS group than in those of the BS group (*p* < 0.01).

### 2.4. Effects on Natural Killer Cells’ Activity

**Figure 3:**
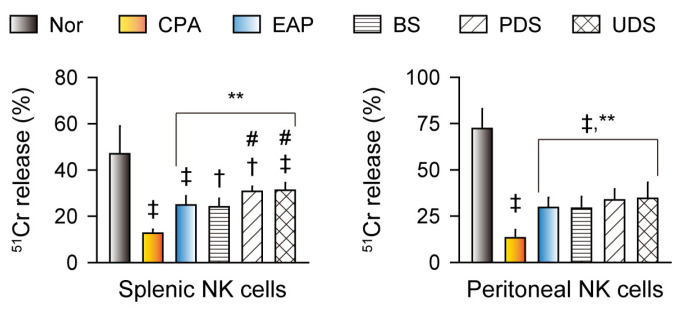
Natural killer (NK) cells’ activities. Splenic and peritoneal activities of NK cells in the cyclophosphamide (CPA)-induced immunosuppressive model after oral administration of Korean seawaters. The CPA model was administered an exopolymer from *Aureobasidium pullulans* SM-2001 (EAP) or three samples of Korean seawaters, namely deep seawaters from off the coasts of Pohang (PDS) and Uljin (UDS), and seawater from off the coast of Boryeong (BS). Values are expressed as the means ± SDs of 10 samples. ‡: *p* < 0.01 and †: *p* < 0.05 compared with the normal control (Nor); **: *p* < 0.01 compared with the CPA model control (CPA); #: *p* < 0.05 compared with the BS group.

Natural killer (NK) cells exert cytotoxic functions in innate immunity, and enhance adaptive immune responses by producing various cytokines, including interferon (IFN) γ and tumor necrosis factor (TNF) α, to stimulate macrophages and dendritic cells as antigen-presenting cells [24]. The splenic and peritoneal activities of NK cells were reduced in the CPA control group compared with the normal control; however, they were increased in the EAP, BS, PDS, and UDS groups compared with the CPA control (*p* < 0.01, Figure 3). The splenic activities of NK cells were significantly higher in the PDS and UDS groups than in the BS group (*p* < 0.01).

### 2.5. Effects on Cytokine Production and Gene Expression

**Figure 4:**
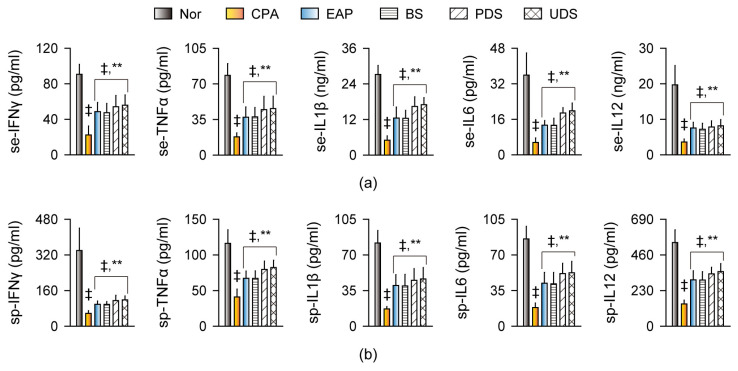
Cytokine production. (**a**,**b**) Serum (se) and splenic (sp) levels of interferon (IFN) γ, tumor necrosis factor (TNF) α, interleukin (IL) 1β, IL6, and IL12 in the cyclophosphamide (CPA)-induced immunosuppressive model after oral administration of Korean seawaters. The CPA model was administered an exopolymer from *Aureobasidium pullulans* SM-2001 (EAP) or three samples of Korean seawaters, namely deep seawaters from off the coasts of Pohang (PDS) and Uljin (UDS), and seawater from off the coast of Boryeong (BS). Values are expressed as the means ± SDs of 10 samples. ‡: *p* < 0.01 compared with the normal control (Nor); **: *p* < 0.01 compared with the CPA model control (CPA).

**Figure 5:**
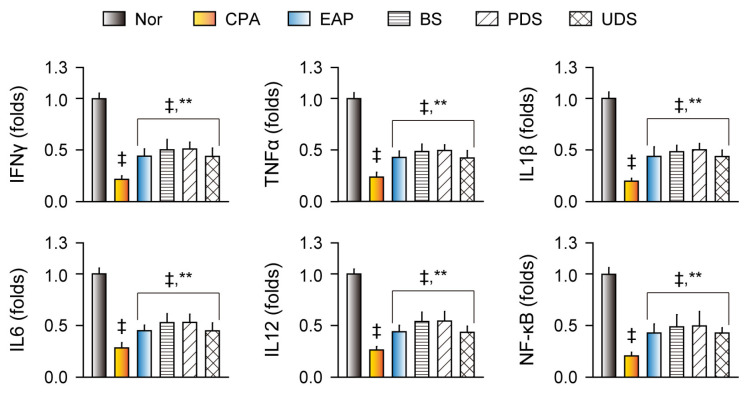
Gene expression of cytokines and the relevant transcription factors. Relative gene expression of the cytokines (IFNγ, TNFα, IL1β, IL6, and IL12) and nuclear factor kappa light chain enhancer of activated B cells (NF-κB) in the cyclophosphamide (CPA)-induced immunosuppressive model after oral administration of Korean seawaters. The CPA model was administered an exopolymer from *Aureobasidium pullulans* SM-2001 (EAP) or three samples of Korean seawaters, namely deep seawaters from off the coasts of Pohang (PDS) and Uljin (UDS), and seawater from off the coast of Boryeong (BS). Values are expressed as the means ± SDs of 10 samples. ‡: *p* < 0.01 compared with the normal control (Nor); **: *p* < 0.01 compared with the CPA model control (CPA).

Immunostimulatory effects were examined by the serum and splenic levels of IFNγ, TNFα, IL1β, IL6, and IL12 (Figure 4). Both serum and splenic cytokine levels were significantly reduced in the CPA control group compared with the normal control; however, they were increased in the EAP, BS, PDS, and UDS groups compared with the CPA control (*p* < 0.01, Figure 4a,b). In addition, splenic gene expressions of the cytokines and nuclear factor kappa light chain enhancer of activated B cells (NF-κB) were downregulated in the CPA control group compared with the normal control; however, they were upregulated in the EAP, BS, PDS, and UDS groups compared with the CPA control (*p* < 0.01, Figure 5).

### 2.6. Histopathological Changes in the Lymphoid Organs

**Figure 6:**
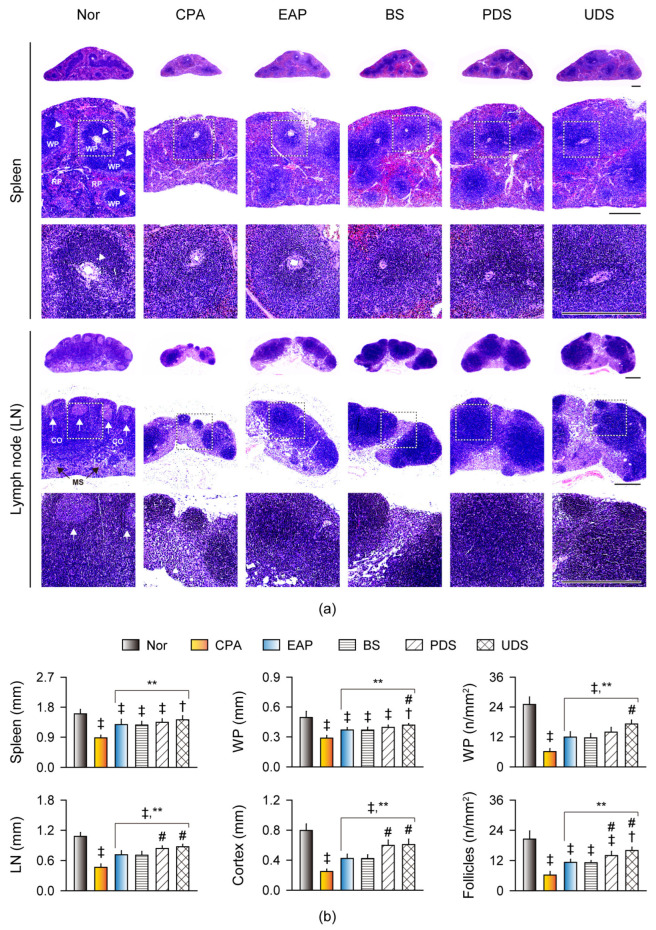
Histopathological changes in the spleen and lymph nodes. (**a**) Representative images of the spleen and lymph nodes (LN) in the cyclophosphamide (CPA)-induced immunosuppressive model after oral administration of Korean seawaters. The CPA model was administered an exopolymer from *Aureobasidium pullulans* SM-2001 (EAP) or three samples of Korean seawaters, namely deep seawaters from off the coasts of Pohang (PDS) and Uljin (UDS), and seawater from off the coast of Boryeong (BS). Dotted boxes are shown in high magnification below. WP and RP represent the splenic white and red pulps, respectively, and the arrowhead indicates the central arteriole. CO and MS in LN represent the cortex and medullary sinus, respectively, and arrows indicate the lymphoid follicles. Scale bars = 400 μm. (**b**) Histomorphometric analyses of the thickness of the spleen, the thickness and number of the splenic WP, the thickness of the LN and its cortex, and the number of lymphoid follicles. Values are expressed as the means ± SDs of 10 samples. ‡: *p* < 0.01 and †: *p* < 0.05, compared with the normal control (Nor); **: *p* < 0.01, compared with the CPA model control (CPA); #: *p* < 0.05, compared with the BS group.

The histopathological changes involved in immunostimulation were examined in the spleen and lymph nodes (Figure 6). The CPA control exhibited evident atrophic changes in the lymphoid organs, with reduced numbers and sizes in the splenic white pulps surrounding the central arterioles, as well as the lymph nodes’ secondary follicles (Figure 6a). However, the atrophic changes were mild in the EAP, BS, PDS, and UDS groups, and the lymphoid cells in the splenic white pulp and follicles of the lymph node (similar to primary follicles) were increased. Histomorphometric analyses revealed significant reductions in the thickness of the spleen, the thickness and number of splenic white pulps, the thickness of the lymph node and its cortex, and the number of follicles of the lymph node in the CPA control group compared with the normal control; however, these parameters were significantly increased in the EAP, BS, PDS, and UDS groups compared with the CPA control (*p* < 0.01, Figure 6b). Additionally, the thickness and number of splenic white pulps were increased in the UDS group compared with the BS group, and the thickness of the lymph node and its cortex, as well as the number of the lymph nodes’ follicles, were increased in the PDS and UDS groups compared with the BS group (*p* < 0.01).

## 3. Discussion

Consistent with previous studies [21,22,25], CPA-induced immunosuppression resulted in reduced weight of the spleen and lymph nodes, accompanied by inhibition of NK cells’ activity and cytokine production. Histopathological analyses supported immunosuppression, showing evident atrophic changes with a depletion of immune cells in the lymphoid organs. However, oral administration of the Korean mineral-rich seawaters, particularly PDS and UDS, significantly improved the immunosuppression. This is the first study to report the immunostimulatory effects of these seawaters in a CPA-induced mouse model. No side effects such as changes in body weight, vomiting, or diarrhea were observed in any of the groups with or without the seawaters, similar to other studies [26,27,28]. In addition, no cytotoxicity was found in the primary splenocytes treated with the seawaters at a concentration similar to the oral dose. These results suggest the potential use of Korean mineral-rich seawaters as a safe alternative immunostimulant.

The treatments of BS, PDS, and UDS increased cytokine production, along with upregulation of NF-κB, a key transcription factor for the expression of proinflammatory genes [29]. It can be presumed that the Korean seawaters may stimulate immune responses by engaging both the innate and adaptive immune cells as the main sources for cytokine production through an NF-κB-driven pathway [30]. Indeed, the seawaters increased NK cells’ activities in vivo and the ConA- and LPS-induced lymphoproliferation ex vivo, resulting in improvements in the CPA-induced atrophic changes with lymphoproliferation in the splenic white pulp and follicles of the lymph nodes. The elevated levels of TNFα, IL1β, and IL6, as major proinflammatory cytokines of the acute phase response in innate immunity, may further promote actions of innate (e.g., NK cells, monocytes/macrophages, etc.) and adaptive immune cells (e.g., lymphocytes) [30,31]. Furthermore, IFNγ may also enhance the activities of NK cells and macrophages with complex effects on lymphocytes’ functions, and IL12 may stimulate the production of TNFα and IFNγ, and mediate the differentiation of T cells into T helper type 1 (Th1) cells. Clinical intake of DS has been reported to improve allergic skin responses by restoring mineral imbalances in atopic eczema/dermatitis syndrome [16], and by reducing levels of IgE and Th2 cytokines (IL4, IL6, and IL13) in allergic rhinitis [17]. Avène spring water, as a mineralized water, has shown proliferative effects in lymphocytes isolated from atopic dermatitis (AD) patients, along with increases in Th1 cytokines (IFNγ and IL2) and reductions in Th2 cytokines [32]. Similarly, topical application of DS in the AD mouse model improved the disease, involving reduced serum levels of proinflammatory cytokines (TNFα, IL1β, and IL6) and Th2-dependent cytokines (IL4 and IL10), but no changes in the levels of IFNγ and IL2 after the induction of AD or application of DS [33]. It is known that AD is characterized by a reduction in the Th1 cell-mediated response and an increase in Th2-driven IgE hypersensitivity. Th1 and Th2 cells and their cytokines have antagonistic effects that mutually inhibit the other’s immune function [30]. This suggests that micronutrients contained in the Korean seawaters may preferentially enhance the Th1-related immune response or regulate the Th1/Th2 imbalance. Further studies are needed to clarify the immune modulation in the long-term kinetics.

Mineral contents in DS have been reported to vary depending on the geological locations and water processes including filtration, reverse osmosis, and concentration [33,34,35,36]. Compared with the mineral contents of other DS sources, the Korean seawaters contained similar or higher levels of Fe, Cu, F, and Se, while they had lower levels of other minerals (e.g., Na, K, Cl^−^, Mg, etc.). The PDS and UDS contained slightly higher levels of Ca, Cr, and Zn than the BS, but lower levels of F, aluminum, and barium. The immunomodulating roles of the abundant minerals in the Korean seawaters have been reported as follows [11,12,37]: Fe stimulates the polarization of macrophages, recruitment of neutrophils, NK cells’ activity, and the lymphocytes’ function and differentiation; Cu and Zn contribute to the maintenance of immune competence by enhancing innate and adaptive immune cells and producing cytokines and antibodies; Se is involved in enhancing T cell-mediated immune responses; Ca ions provide the cytosolic signals required for immune responses in stimulating cytotoxic T cells and NK cells and promoting immune cells’ recruitment and function [38]; low levels of F and Cr act as an adjuvants for systemic immunity and a stimulant for the macrophages and the humoral immune response, respectively, while high levels can have adverse consequences on the immune system [39,40]. Furthermore, significant antioxidant actions of Ca, Cr, Cu, Fe, Se, and Zn may prevent the impairment of the immune response and inflammatory progression [6,12,13]. However, supplementation with Zn and Se can inhibit the production of proinflammatory cytokines as a negative regulator of the NF-κB signaling pathway, and overdoses of Cu and Fe can increase the growth of microbial pathogens [11]. In this context, the immunostimulatory effects of Korean seawaters may be involved in the interconnected effects of the combined actions of several minerals on both innate and adaptive immunity. Trace mineral deficiency is not common or considered a major problem in humans; however, Ca, Fe, Zn, and Se are among the most common micronutrient deficiencies in the world [8,14]. Although further studies are needed to clarify the detailed mechanisms of the minerals’ actions, micronutrients, including the minerals in the PDS and UDS, may contribute to immunostimulatory effects by restoring mineral deficiencies.

Mineral deficiency can result from insufficient intake, impaired absorption, or the types of food consumed. Western-style or instant foods and vegan diets may be insufficient to supply vital minerals, and consumption of phytate-rich foods can inhibit the absorption of certain minerals (e.g., Ca, Fe, and Zn) [41]. The effects of minerals on immunomodulation have been reported from balneotherapy in Dead Sea in AD patients [42] and in mineral-rich seawaters and groundwater in an AD animal model [20,43]. People often consume commercially available mineral water, with the global market size and consumption expected to increase further [44]. While water with high hardness may have adverse effects on the human body, DS, with hardness ranging from 0 to 1500 mg/L, has been found to have no cytotoxicity and no damage to the liver and kidney [13,45]. The Korean seawaters used showed a hardness of about 340–352 mg/L, and contained few hazardous chemicals such as arsenic, lead, cadmium, and mercury. The PDS and UDS may especially have minimal to no bacterial activity due to their deep locations far from solar radiation, which may preserve abundant unknown nutrients [13]. This suggests that the Korean mineral-rich seawaters may provide essential minerals involved in immunostimulation as a nutrient source. Furthermore, DS containing several minerals (i.e., Ca, Mg, and Zn) has shown anticancer effects by inhibiting the transforming growth factor β and Wnt5a signaling that is responsible for augmenting the Th2-driven pathway [46]. Considering that CPA, a widely used anticancer drug, commonly induces immunosuppression as a side effect [47], the Korean seawaters could be safe candidates for concurrent administration with anticancer drugs. These results provide useful information for the use of Korean mineral-rich seawaters as alternative immunostimulants under immunosuppressive conditions.

## 4. Materials and Methods

### 4.1. Korean Seawaters and Reagents

The Korean Institute of Ocean Science and Technology (Busan, Republic of Korea) provided the seawaters and analyzed the mineral contents. Briefly, the BS, PDS, and UDS were collected from depths of 50 m, 200 m, and 250 m in the sea located at the latitude and longitude of 36.30N 125.90E, 35.59N 129.55E, and 36.95N 130.10E, respectively. After the seawaters were filtered using a microfilter system (Synopex INC, Pohang, Republic of Korea), the main minerals (Na, Mg, Ca, and K) and anions were analyzed using atomic absorption spectrophotometry and ion chromatography, respectively. Other trace minerals were determined using inductively coupled plasma mass spectrometry, as described elsewhere [48,49]. Hardness was calculated using Equation (1):

Hardness (mg/L) = Mg (mg/L) × 4.1 + Ca (mg/L) × 2.5,(1)

The seawaters were diluted in DW to contain 0.9% NaCl, the physiological isotonic level, and stored at 4 °C until use. EAP containing 13% β-1,3/1,6-glucan (Polycan^TM^) was purchased from Glucan Corp. (Busan, Republic of Korea), and reagents including CPA, ConA, LPS, and 3-(4,5-dimethylthiazol-2-yl)-2,5 diphenyl tetrazolium bromide (MTT) were purchased from Sigma-Aldrich (St. Louis, MO, USA).

### 4.2. Animal Treatments

All animals were treated according to international regulations on the usage and welfare of laboratory animals, and the experimental protocol was approved by the Institutional Animal Care and Use Committee of Daegu Haany University (Approval No. DHU2022-068). Six-week-old male SPF/VAF CrlOri:CD1 (ICR) mice were obtained from Orient-Bio Inc. (Seongnam, Republic of Korea), and housed in a temperature- (20–25 °C) and humidity-controlled (50–55%) room, with a 12:12 h light/dark cycle. Feed and water were supplied ad libitum. They were acclimatized for a week, and then divided into six groups (*n* = 10 per group) on the basis of body weight. The mice were administered treatments through oral gavage once a day for 42 days at a volume of 10 mL/kg as follows: one normal group with DW (normal control) and five CPA-induced model groups with DW (CPA control), EAP at 200 mg/kg (EAP), and three kinds of Korean seawaters (BS, PDS, and UDS). The dose of EAP was determined as described previously [21,22]. The CPA model groups were injected intraperitoneally with CPA at 150 and 100 mg/kg in saline on Days 40 and 42 post-administration, respectively, at a volume of 10 mL/kg. The normal control was injected with saline alone. Body weight was measured once a week. Blood was collected under anesthesia using a mixture of 70% N_2_O and 28.5% O_2_ with 2–3% isoflurane, 24 h after the second injection of CPA. Animals were then euthanized using CO_2_ gas, and the spleen and left submandibular lymph nodes were sampled.

### 4.3. Preparation of Primary Splenocytes

Primary splenocytes were isolated from the mice of all groups, as described previously [22]. A part of the spleen sample (20 mg) was minced into fragments in RPMI-1640 (Gibco BRL, Grand Island, NY, USA) supplemented with 10% fetal bovine serum (Gibco BRL), 100 U/mL penicillin, 100 μg/mL streptomycin, and 2 mM glutamine (Sigma-Aldrich). Major tissue aggregates were removed through a 200 μm mesh nylon screen (Sigma-Aldrich), and the erythrocytes were lysed with a 0.83% ammonium chloride solution. After washing three times, the viability of splenocytes was assessed by staining with trypan blue using an automated cell counter (Countess C10281; Invitrogen, Carlsbad, CA, USA). The splenocytes were maintained in a 37 °C humidified incubator with 5% CO_2_.

### 4.4. Cell Viability and Proliferation Assays in Primary Splenocytes

For the cell viability assay, the normal control-derived splenocytes were cultured in a 96-well plate (1 × 10^5^ cells/well) for 24 h and then treated with the Korean seawaters at seven dose ranges from 0.01% to 10% in the culture medium for 72 h. The cells were incubated with 2.5 mg/mL MTT for 4 h at 37 °C and lysed in dimethyl sulfoxide. Absorbance was measured at 570 nm using a microplate reader (Sunrise; Tecan, Männedorf, Switzerland). For the proliferation assay, splenocytes isolated from the mice of all groups were cultured in a 96-well plate (1.2 × 10^4^ cells/mL) for 24 h and treated with ConA at 5 μg/mL or LPS at 10 μg/mL for 48 h. The cells were incubated with 5 μg/mL MTT for 4 h at 37 °C and dissolved with 0.1 N hydrogen chloride in 10% sodium dodecyl sulfate. Absorbance was measured at 560 nm using a microplate reader. Six independent assays for cell viability and proliferation were conducted.

### 4.5. Assessments of NK Cell Activity

Splenic and peritoneal NK cell activities were measured using a standard ^51^Cr release assay, as described previously [22]. The spleen (10–20 mg) was homogenized in an RPMI-1640 medium, and the peritoneal cells were collected by repeated intraperitoneal washes with RPMI-1640. The spleen homogenates and peritoneal resuspension were disrupted by maceration and washing with RPMI-1640 through a wire mesh (Mesh No. 100, Sigma-Aldrich). After centrifugation, the cell pellets were incubated in 1% ammonium oxalate on ice for 10 min to remove RBCs and washed twice with Hanks Balanced Salt Solution (Gibco BRL). The splenocytes and peritoneal cells were cultured overnight in Dulbecco’s Modified Eagle Medium (Invitrogen, Grand Island, NY, USA) with or without recombinant IL2 at 1000 IU/mL (Proleukin Chiron, Emeryville, CA, USA) and in a complete culture medium (Sigma-Aldrich), respectively. HTLA-230 neuroblastoma target cells, which specifically react with NK cells, were labeled with Na_2_^51^CrO_4_ (100 μCi/1 × 10^6^ cells) (ICN Biomedicals, Asse, Belgium) for 2 h and then incubated with the splenocytes or peritoneal cells as effector cells for 6 h at 37 °C. The ratios of effector to target cells were 100:1 for splenocytes and 10:1 for peritoneal cells. The supernatants were collected, and released radioactivity was assessed using a Cobra 5002 gamma counter (Canberra Packard, Meriden, CT, USA). The percentage of NK cells’ activity was calculated using Formula (2)

% specific ^51^Cr release (NK cell activity) = [(Exp − S)/(M − S)] 100%, (2)

where ‘Exp’ represents the released ^51^Cr value, and ‘S’ and ‘M’ are the spontaneous and maximum values of ^51^Cr, respectively.

### 4.6. Assessments of Serum and Splenic Cytokine Levels

Blood (about 0.5 mL) was centrifuged at 14,000× *g* for 10 min at 4 °C, and the resulting serum was collected. The spleen sample (about 10 mg) was homogenized in phosphate-buffered saline-based lysis buffer (2 mM phenylmethylsulfonyl fluoride and 1 mg/mL of aprotinin, leupeptin, and pepstatin A) using a bead beater (Taco^TM^Pre, Gene Research Biotechnology Corp., Taichung, Taiwan) and an ultrasonic cell disruptor (KS-750, Madell Technology Corp., Ontario, CA, USA). The serum and splenic levels of IFNγ, TNFα, IL1β, IL6, and IL12 were assessed using enzyme-linked immunosorbent assay kits, according to the manufacturer’s instructions (for IFNγ, #MBS2500105; for TNFα, #MBS825075; for IL1β, #MBS175967; for IL6, #MBS2508516; for IL12, #MBS2510359, all from MyBioSource, San Diego, CA, USA). Absorbance was measured at 450 nm under the standard curves using a microplate reader. All assays were performed in duplicate.

### 4.7. Real-Time Reverse Transcription Polymerase Chain Reaction (RT-PCR)

The relative gene expressions of IFNγ, TNFα, IL1β, IL6, IL12, and NF-κB were examined in the spleen tissues, as described previously [22]. RNA was extracted using TRIzol reagent (Invitrogen, Carlsbad, CA, USA), and its concentration and quality were determined by a CFX96^TM^ Real-Time System (Bio-Rad, Hercules, CA, USA). To remove contaminating DNA, samples were treated with a DNA-free DNA Removal kit (#AM1906, Thermo Fisher Scientific Inc., Rockford, IL, USA). RNA was reverse-transcribed using the High-Capacity cDNA Reverse Transcription Kit (#4368813, Thermo Fisher Scientific Inc.), according to the manufacturer’s instructions. The cDNA products were combined with specific primers listed in Table 2. Amplification was conducted under the following conditions: 10 min at 94 °C, and 39 cycles of 15 s at 94 °C, 20 s at 57 °C, and 30 s at 72 °C, using the ABI Step One Plus Sequence Detection System (Applied Biosystems, Foster City, CA, USA). The genes’ expression levels were normalized to that of glyceraldehyde 3-phosphate dehydrogenase (GAPDH) as an internal control gene, and calculated relative to the normal control, using the comparative threshold cycle method [50].

### 4.8. Histopathological Analysis

Some parts of the spleen and lymph nodes were fixed in 10% neutral buffered formalin and embedded in paraffin. The samples were serially sectioned at a thickness of 3 μm and stained with hematoxylin and eosin. Histomorphometric analyses were performed as described previously [21,22,25]. In the spleen, the thickness from the anterior apex to the center of the posterior border, and the number and size of the splenic white pulps were measured. In the lymph node, the thickness of the central region and cortex, and the number of lymphoid follicles were measured. These measurements were performed using an image analyzer (*i*Solution FL ver 9.1, IMT *i*-Solution Inc., Vancouver, BC, Canada) by a histopathologist blinded to the experimental groups.

### 4.9. Statistical Analyses

All data were expressed as the means ± standard deviations (SDs) of six independent experiments ex vivo and ten samples in vivo. The normal distribution of the variables and the homogeneity of variances were examined by the Kolmogorov–Smirnov and Levene tests, respectively. Given the normal distribution of the variables, the data were examined by one-way ANOVA. The kinetic changes in body weight were examined by two-way ANOVA with the main factors for the groups and time-points, treating the time-points as a repeated measure. Multiple comparisons were conducted using Tukey’s HSD and Dunnett’s T3 post hoc tests in cases of equal and non-equal variances, respectively. A *p*-value of less than 0.05 was considered statistically significant.

## Figures and Tables

**Table 1:** Mineral contents in Korean seawaters.

Major (ppm)	BS	PDS	UDS	Trace (ppb)	BS	PDS	UDS
Na (sodium)	3597.5	3557.2	3477.6	Ni (nickel)	86.4	88.4	86.3
Cl^−^ (chloride)	6544.4	6499.6	6379.9	Li (lithium)	32.3	31.9	32.8
SO_4_^2−^ (sulfate)	682.9	687.6	672.8	Rb (rubidium)	28.4	29.0	29.0
K (potassium)	120.5	118.3	117.8	Cu (copper)	19.3	28.3	18.7
Ca (calcium)	120.5	137.4	132.8	Co (cobalt)	15.3	16.0	15.6
Br^−^ (bromide)	73.4	73.6	73.1	Zn (zinc)	13.5	16.1	15.4
NO_3_^−^ (nitrate)	66.9	64.3	61.8	Se (selenium)	13.2	12.7	13.1
F^−^ (fluoride)	36.4	32.4	32.4	V (vanadium)	11.4	11.5	11.5
Sr (strontium)	1.8	1.9	1.8	As (arsenic)	5.2	5.0	4.9
Mg (magnesium)	1.4	1.5	1.3	Ba (barium)	3.5	1.5	1.8
B (boron)	1.2	1.2	1.2	Mo (molybdenum)	3.1	3.1	3.1
Fe (iron)	0.4	0.4	0.4	Al (aluminum)	2.3	0.7	1.6
Ge (germanium)	0.3	0.3	0.3	Cr (chromium)	0.5	0.6	0.6
Ti (titanium)	0.2	0.3	0.3	Mn (manganese)	0.4	0.5	0.4

Two samples of Korean deep seawaters were collected from the East Sea, off the coasts of Pohang (PDS) and Uljin (UDS), respectively, and another seawater sample was collected from the West Sea, off the coast of Boryeong (BS). Minerals containing more than 0.5 ppb (μg/L) in the seawaters are listed and divided into major and trace elements based on an amount of 0.1 ppm (mg/L).

**Table 2:** Primer sequences for real-time reverse transcription polymerase chain reaction.

Targets (GenBank IDs)	Sequence (5′–3′)
NF-κB (NM008689)	Forward: CAATGGCTACACAGGACCA
	Reverse: CACTGTCACCTGGAACCAGA
INFγ (NM008337)	Forward: GTTACTGCCACGGCACAGTCATTG
	Reverse: ACCATCCTTTTGCCAGTTCCTCCAG
TNFα (NM013693)	Forward: CCTGTAGCCCACGTCGTAGC
	Reverse: TTGACCTCAGCGCTGAGTTG
IL1β (NM008361)	Forward: AGCTGTGGCAGCTACCTGTG
	Reverse: GCTCTGCTTGTGAGGTGCTG
IL6 (NM031168)	Forward: TCTTGGGACTGATGCTGGTGAC
	Reverse: GCAGCTCCCTCTTGTTGT
IL12 (NM008354)	Forward: CAGGTGTCTTAGCCAGTCC
	Reverse: CATAACGCACTAGGTTTGCCGA
GAPDH (NM008084)	Forward: CATCTTCCAGGAGCGAGACC
	Reverse: TCCACCACCCTGTTGCTGTA

INF, interferon; GAPDH, glyceraldehyde 3-phosphate dehydrogenase; IL, interleukin; NF-κB, nuclear factor kappa light chain enhancer of activated B cells; TNF, tumor necrosis factor.

## Data Availability

Data are contained within the article.
